# Descriptive Analysis of Cerebral Arterial Vascular Architecture in Dromedary Camel (*Camelus dromedarius*)

**DOI:** 10.3389/fnana.2019.00067

**Published:** 2019-07-05

**Authors:** Ahmad Al Aiyan, Preetha Menon, Adnan AlDarwich, Fatema Almuhairi, Shaikha Alnuaimi, Asma Bulshawareb, Moneeb Qablan, Safa Shehab

**Affiliations:** ^1^Department of Veterinary Medicine, College of Food and Agriculture, United Arab Emirates University, Abu Dhabi, United Arab Emirates; ^2^Department of Anatomy, College of Medicine and Health Sciences, United Arab Emirates University, Abu Dhabi, United Arab Emirates

**Keywords:** brain, Circle of Willis, corrosion cast, dromedary, camel, rostral epidural rete mirabile

## Abstract

The artiodactyl brain has multiple levels of vascular pooling and the rostral epidural rete mirabile (RERM) at its base. The current study is the first of its kind to precisely demonstrate the arterial vasculature of the dromedary brain, utilizing a new casting method with colored latex and epoxy paint. In total, 35 freshly slaughtered dromedary heads were injected with colored latex or colored epoxy paint prior to dissection in order to reveal cerebral vasculature; Ten processed heads were chemically digested with 5% potassium hydroxide to obtain hard casts of cerebral arteries and anastomosing structures. The outcomes of this study ascertain the distinct vascular features of dromedaries that set them apart from other artiodactyls. In addition to the RERM, the dromedary possesses a well-developed ophthalmic and chiasmatic rete. The dromedary is similar to giraffe, goat, cat and pig in the contribution of middle meningeal artery to the rete mirabile; however, dromedaries have several arteries emerging directly from the cerebral arterial circle that supply the choroid plexus and pineal gland. Additionally, dromedaries exhibit a dominant basilar system that dominates the blood supply to the medulla oblongata, pons, and cerebellum. In our study, we were able to graphically prove the lack of connection between the areas supplied by vertebrobasilar system and carotid system in the dromedary. Furthermore, the vertebral artery does not branch into the basilar artery; instead, it acts as a contributing vessel to the ventral spinal artery that later fuse to form the basilar artery. This study employed the new casting method to illustrate a new arterial source to RERM and the various anastomoses among arterial sources supplying the brain in the dromedary. These anastomoses play an important role in maintaining an uninterrupted cerebral blood supply, decreasing the vulnerability of the fragile brain against ischemia and stroke, as well as, play an important role in maintaining blood pressure and flow in long-necked dromedaries when they raise or lower their heads.

## Introduction

The cerebral vasculature is characterized by a complex network of collateral circulation. Cerebral tissue could be supplied by multiple blood vessels from different afferent sources. This multi-sourced contribution decreases the vulnerability of the fragile brain, which acts as an effective insurance against ischemia and stroke because blockage in one set of afferent arteries is countered by a backup supply of oxygenated blood from its collateral artery originating from a different parent (Chuang et al., 2009; Hoffmann et al., 2014).

Most mammals can maintain an uninterrupted cerebral blood supply through three unique architectures of afferent blood vessels. The first system that ensures incessant supply includes dual or multiple sources of arterial blood. The heart pumps oxygenated blood to the brain through multiple routes. The main route is through the carotid system, wherein blood reaches the brain through five branches and sub-branches of the common carotid artery, namely, the internal carotid, maxillary, external ophthalmic, middle meningeal, and occipital arteries (Lesbre, 1903; Daniel et al., 1953; Smuts and Bezuidenhout, 1987; Ocal et al., 1999; Kiełtyka-Kurc et al., 2014; Jerbi et al., 2016; Khairuddin et al., 2017). The less dominant, but equally significant system is the vertebrobasilar system, wherein oxygenated blood travels through the vertebral artery that climbs up along the vertebral column and contributes to the basilar artery that enters the cranium to supply the brain from the posterior end of the foramen magnum (Zguigal, 1988; Ocal et al., 1999).

The second adaptive strategy to prevent ischemia is to pool the blood coming from different afferent systems before supplying it to the brain. This is seen in the cerebral arterial circle, popularly known as the Circle of Willis. The arterial circle allows blood from the carotid system and vertebrobasilar system to mix through a complex set of anastomosing blood vessels at the floor of the cranial cavity (Smuts and Bezuidenhout, 1987; Ocal et al., 1999; Kiełtyka-Kurc et al., 2014). The blood vessels emerging from the arterial circle then supply different brain structures. This pooling of blood is also found in arterial shunts other than those in the arterial circle (Kiełtyka-Kurc et al., 2014). Such individual anastomoses in sites other than the cranial floor will be discussed later.

The brain stem is supplied by the basilar artery in addition to the pooled blood from the arterial circle. This added protection to the brain stem could be attributed to its involvement in maintaining the brain and vital cardio-respiratory functions (Kiełtyka-Kurc et al., 2014; Terlouw et al., 2016; Sorby-Adams et al., 2018).

In addition to the pooled blood from multiple sources, artiodactyls and carnivores have an extensive network of anastomosing arteries known as the rostral epidural rete mirabile (RERM) (Lesbre, 1903; Zguigal, 1988; García-Villalón et al., 1989; Ocal et al., 1998; Zdun et al., 2013; Sorby-Adams et al., 2018), which not only pools blood from different arteries, but also acts as a reservoir for afferent blood. It is also known to reduce the high cerebral blood pressure from the afferent arteries (Kiełtyka-Kurc et al., 2015). The RERM also dissipates heat from warm arterial blood by virtue of lying in a pool of cooler venous blood. This brings down the temperature of blood reaching the brain (Zguigal, 1988; Samara et al., 2013; Deepthi et al., 2016; Jerbi et al., 2016; O’Brien et al., 2016).

Species that serve as animal models for human neurology, including sheep, cattle, horses, and rats, have been extensively studied (Baldwin and Bell, 1963; Zdun et al., 2013; Parkash and Jain, 2014; Deepthi et al., 2016), and their vasculature and adaptive systems have been well documented. While several studies have explored these anatomical characteristics, they have either lacked detailed description or did not account for the wide variations in camel vasculature (Lesbre, 1903; Smuts and Bezuidenhout, 1987; Zguigal and Ghoshal, 1991b; Ocal et al., 1999; Kiełtyka-Kurc et al., 2014; Jerbi et al., 2016; Khairuddin et al., 2017).

The present study tries to improve on the detailed description of arterial vasculature by developing improved casting methods using epoxy paint. This process gave a better three-dimensional representation and could highlight smaller structures that would have been lost with the traditional latex cast and dissection method. Therefore, this study attempted to provide a detailed illustration of the cerebral arteries in the camel, focusing on the afferent arterial systems. This study also tries to record the major variations in vascular design, with a larger sample size of 35 camel heads.

## Materials and Methods

This study adhered to the recommendations by UAEU Research Ethics Committees and was approved by the Animal Research Ethics Committee (A-REC) at the United Arab Emirates University (ERA_2019_5850). The reported experiments comply with the ARRIVE guidelines.

In this study, 35 heads of freshly slaughtered male Omani dromedaries aged 2–6 years were procured from Al Khazna Camel Slaughterhouse (24°06′21.4′′N 55°07′31.6′′E), Abu Dhabi Food Control Authority (ADFCA) and dissected out to map the arterial circulation to the brain. Subjective sampling till data saturation was adopted. The obtained heads included necks with at least two cervical vertebrae. The main method involved injecting the arteries of the head and cranial extremity of the neck with two different diluted solutions (solvent-free colored epoxy paint and colored latex neoprene) *via* cannulas placed in the right and left common carotid arteries. Three camel heads were injected with synthetic latex colored with red acrylic paint mixed with an acid hardener in the ratio 10:1, just before injection. The rest 32 heads were injected with red solvent-free epoxy paint (Gulfguard Epoxy—Color 04E53) mixed with solvent-free hardener (Falco Epoxy Hardener) in the ratio 4:1, just before injection.

Whole camel heads with the first two cranial vertebrae were procured from freshly slaughtered animals for casting. The larger camel heads required ~600 ml of the filler, while the smaller heads required 400 ml. The solutions were injected gradually with hand pressure, using 60-ml syringes, until resistance was felt. To prevent the leakage of the colored solution out of anastomosing vessels, like the vertebral and spinal arteries, they were clamped shut with artery forceps. The heads were then maintained at 5°C for 1–2 days until the arteries had palpable hardness.

The heads were then dissected to remove the surrounding skin and muscles. The roof of the cranium and vertebrae were sliced open with a rotating power saw (DeWalt DWE4001; DeWalt 100 × 0.1 × 16 mm blade), and the cranial cavity was washed using a high-pressure washer. The strong water jet washed away the brain tissues and spinal cord without affecting the epoxy cast fillers. Ten samples were subjected to a slower process of chemical digestion by immersing the craniotomized heads with the first two spinal vertebrae in 5% potassium hydroxide (Caustic Potash-solid 90%, Albemarle, Jordan), which digested all soft tissues but not the epoxy paint and latex in the arteries. This process took 2 weeks for yielding the residual latex casts which could not withstand the high-pressure wash.

After both the processes, we obtained the bony skulls with a network of arteries molded by the epoxy paint or latex. All of the samples were imaged using Sony a7R II at 42-megapixel Fine JPEG setting for acquiring high-resolution images. Using Adobe Photoshop CC 20.0.1^®^, different colors were selectively applied to the different arteries to define each cerebral artery. Both the original image and its color-enhanced counterpart are presented in this study. This color enhancement using Adobe Photoshop CC was performed to highlight the architecture of the different cerebral arteries and branches of the basilar arteries.

## Results

**Figure 1:**
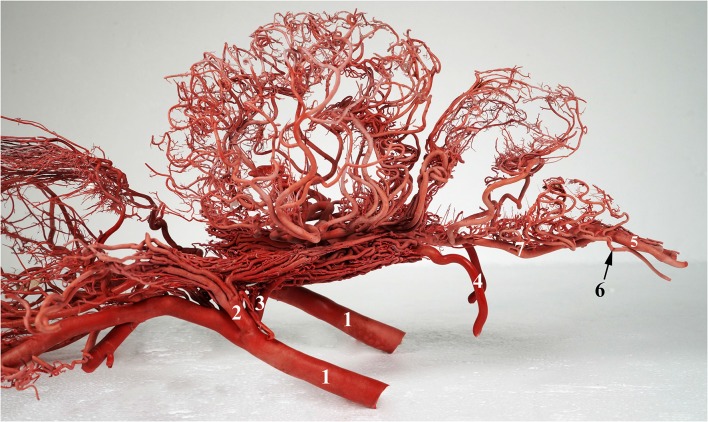
A photograph showing the arterial supply to the dromedary brain (left lateral view). 1, maxillary artery; 2, external ophthalmic artery; 3, rostral branches to the rostral epidural rete mirabile (RERM); 4, internal carotid artery; 5, vertebral artery; 6, lateral branch of the vertebral artery; and 7, basilar artery. Note: middle meningeal artery was not a dominant supplier to RERM in this sample.

**Figure 2:**
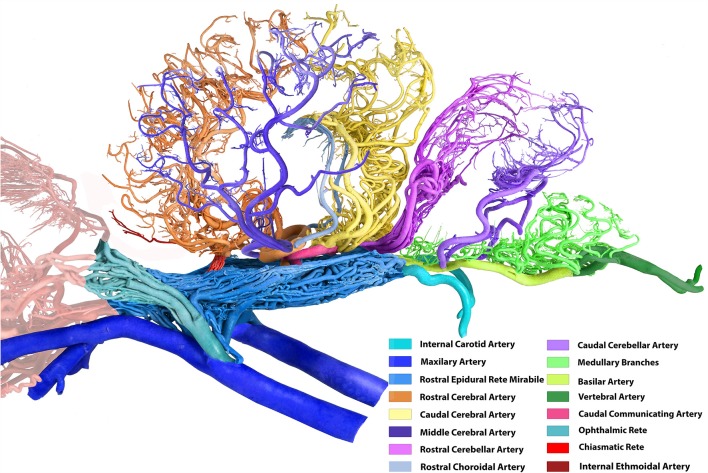
Arterial supply to the brain in the dromedary (left lateral view). Note: middle meningeal artery was not a dominant supplier to RERM in this sample. The middle cerebral artery in the contralateral hemisphere has been removed for better image clarity.

The cerebrum, cerebellum and brain stem are supplied by two arterial systems: the carotid and vertebrobasilar arterial systems. The vertebrobasilar system includes arteries from the vertebral artery and branches of the basilar artery that supply oxygenated blood to the spinal cord, medulla oblongata, pons, cerebellum, and partially, to the midbrain. The carotid arterial system includes all branches and sub-branches emerging from the common carotid artery, namely, the internal carotid, rostral branches to the RERM from the maxillary, external ophthalmic, middle meningeal, and occipital arteries. The carotid arterial system supplies pooled blood to the forebrain and, partially, to the midbrain. This pooling of blood occurs at two levels. The branches of the common carotid artery, namely, the internal carotid, rostral branches to the RERM from the maxillary, middle meningeal, and external ophthalmic arteries, pool their blood in an intricate vascular network called the RERM (Figures 1, 2, 4). This pooled blood from the branches of the common carotid artery mixes with the blood from the branches of the vertebral arteries (mainly the basilar artery) at the base of the brain, in a vascular anastomosing ring called the Circle of Willis or cerebral arterial circle. Herein, we describe these afferent arteries to the different parts of the dromedary brain.

**Figure 3:**
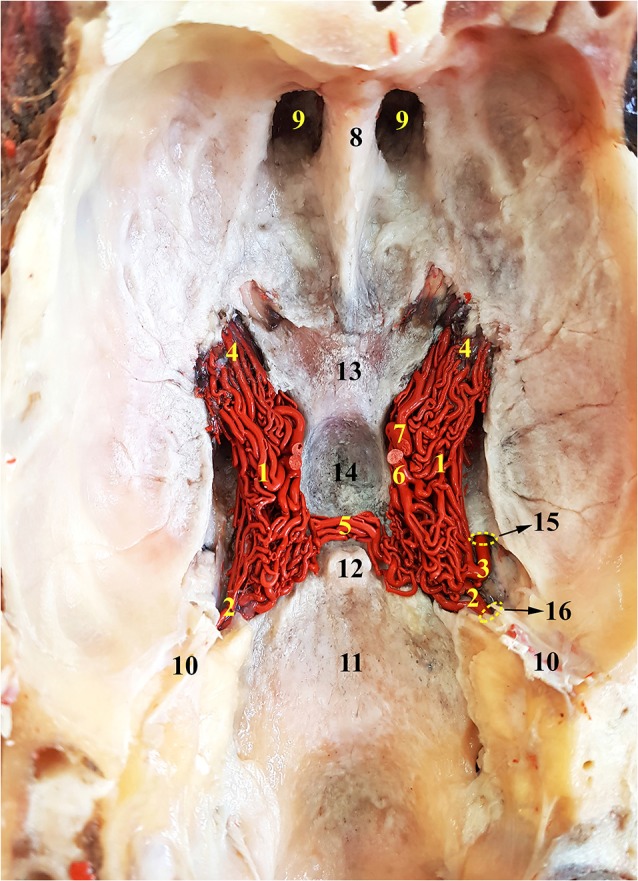
Dorsal view of the RERM of the dromedary. 1, RERM; 2, internal carotid artery; 3, middle meningeal artery; 4, rostral root of the rete; 5, caudal connection of the rete; 6, caudal communicating artery; 7, rostral cerebral artery; 8, crista galli; 9, ethmoid fossa; 10, tentorium cerebelli; 11, caudal cranial fossa; 12, dorsum sellae; 13, chiasmatic sulcus; 14, hypophyseal fossa; 15, internal opening of the foramen ovale; and 16, intracranial entrance of internal carotid artery. Note: middle meningeal artery was a dominant supplier to RERM on the right.

**Figure 4:**
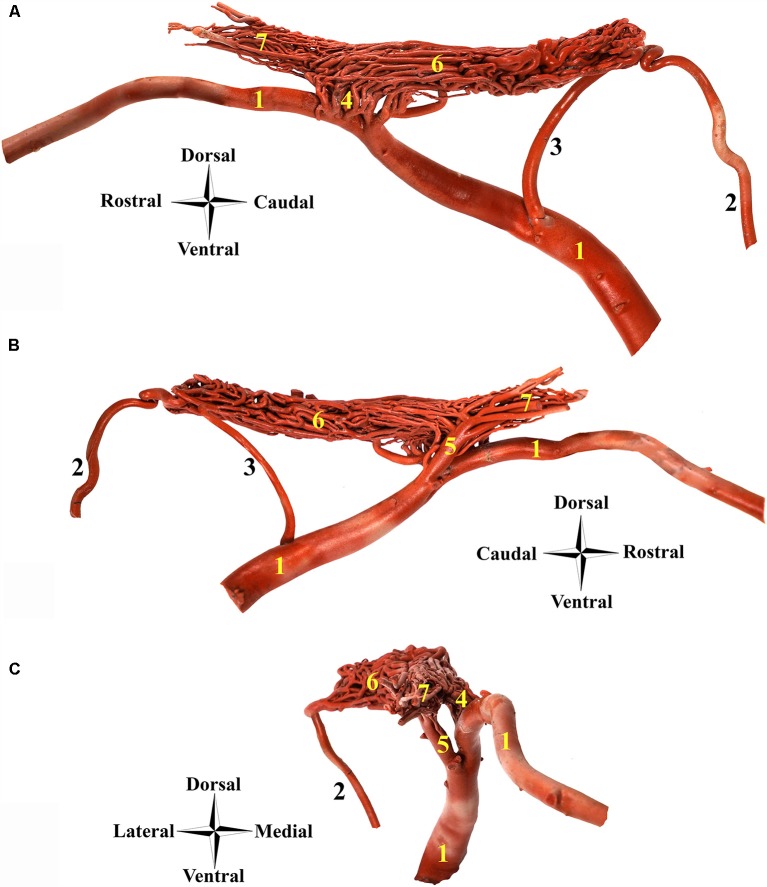
Afferent arteries to the RERM in the dromedary. **(A)** Right medial view, **(B)** right lateral view and **(C)** rostral view; 1, maxillary artery; 2, internal carotid artery; 3, middle meningeal artery; 4, rostral branches to the RERM; 5, external ophthalmic artery; 6, RERM; and 7, ophthalmic rete. Note: this specimen contains a dominant middle meningeal artery contribution to RERM.

The internal carotid, rostral branches to the RERM from the maxillary, middle meningeal, and external ophthalmic arteries enter the cranial cavity and send out branches which meet to form the RERM (Figures 1, 2). These arteries follow their unique routes to enter the cranial cavity.

### Internal Carotid Artery

The internal carotid artery emerges from the common carotid artery at the level ventral to the occipital condyles and travels dorso-rostrally along the lateral aspect of the pharyngeal wall, accompanied by the internal carotid nerve. It then enters the carotid canal through the carotid foramen but does not enter the cranial cavity through the internal opening of the carotid canal. It forms a loop (sigmoid flexure) as it travels rostrally along the tympano-occipital fissure through the ventral petrosal sinus. It enters the cranial cavity through the opening formed by the rostral edge of the petrous and basisphenoid bones (Figure 3). Even before entering the cavernous sinus in the cranial cavity, the internal carotid artery starts dividing into multiple branches. The middle meningeal artery enters the cavernous sinus through the foramen ovale, which lies rostro-lateral to the point of entry of the internal carotid artery (Figures 3, 4). The dividing branches of both the arteries travel together rostrally, parallel to each other, sending out several branches, which anastomose extensively with each other to form the caudal root of the RERM (Figures 3, 4). The internal carotid artery does not send out any branches before entering the cranial cavity.

### Maxillary Artery

The external carotid artery travels dorso-rostrally along the lateral aspect of the pharyngeal wall and continues as the maxillary artery after sending out the facial artery while crossing the stylohyoid bone. The maxillary artery climbs up gradually at 45°, unlike the internal carotid artery which shows a steeper descent (Figures 1, 4). Medial to both pterygoid muscles, it sends out several branches that supply the deep structures lying outside the cranial cavity, before passing through the maxillary foramen towards the infraorbital canal. Due to the absence of the alar canal in the camel, the maxillary artery was always extracanalicular. The maxillary artery sends out the rostral branches that move into the cranium to contribute to the RERM (Figures 1, 4, 7). The rostral branches to the RERM are multiple short branches that vary in number and emerge medially in clusters from the maxillary artery before dividing profusely into long parallel branches. These divided branches double back caudally to enter the cranial cavity through the orbitorotundum foramen. They travel through the foramen caudally before entering the cavernous sinus (Figures 3, 4). These rostral branches then contribute to the rostro-medial portion of the RERM (Figures 4, 7).

**Figure 5:**
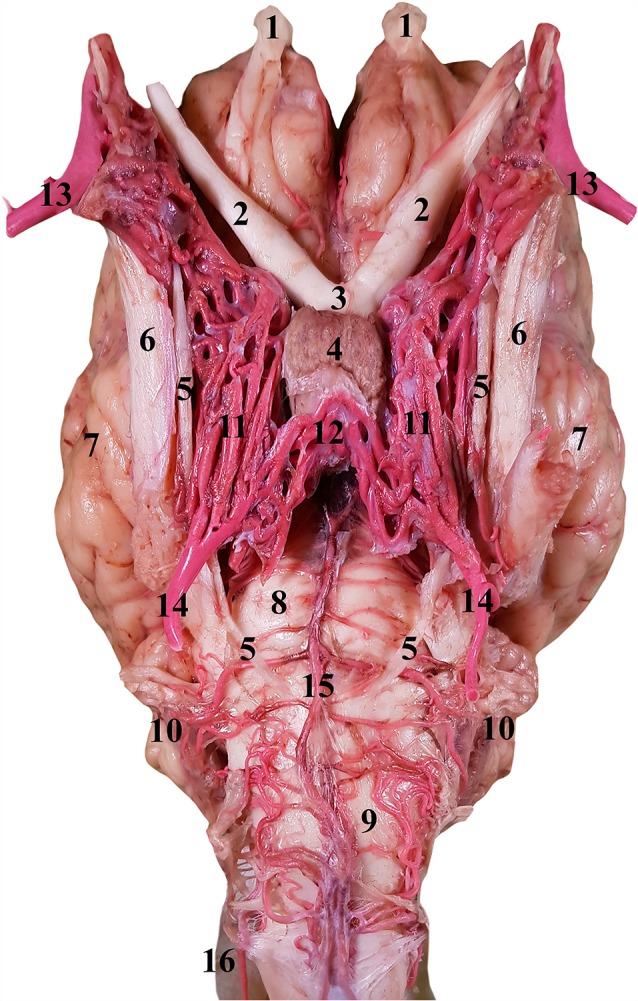
Ventral view of the RERM in the isolated encephalon of the dromedary. 1, Olfactory bulb; 2, optic nerve; 3, optic chiasm; 4, pituitary gland; 5, abducent nerve; 6, trigeminal nerve; 7, left cerebral hemisphere; 8, pons; 9, medulla oblongata; 10, cerebellum; 11, RERM; 12, caudal connection of the rete; 13, maxillary artery; 14, internal carotid artery; 15, basilar artery; and 16, lateral anastomosing branch of the vertebral artery.

**Figure 6:**
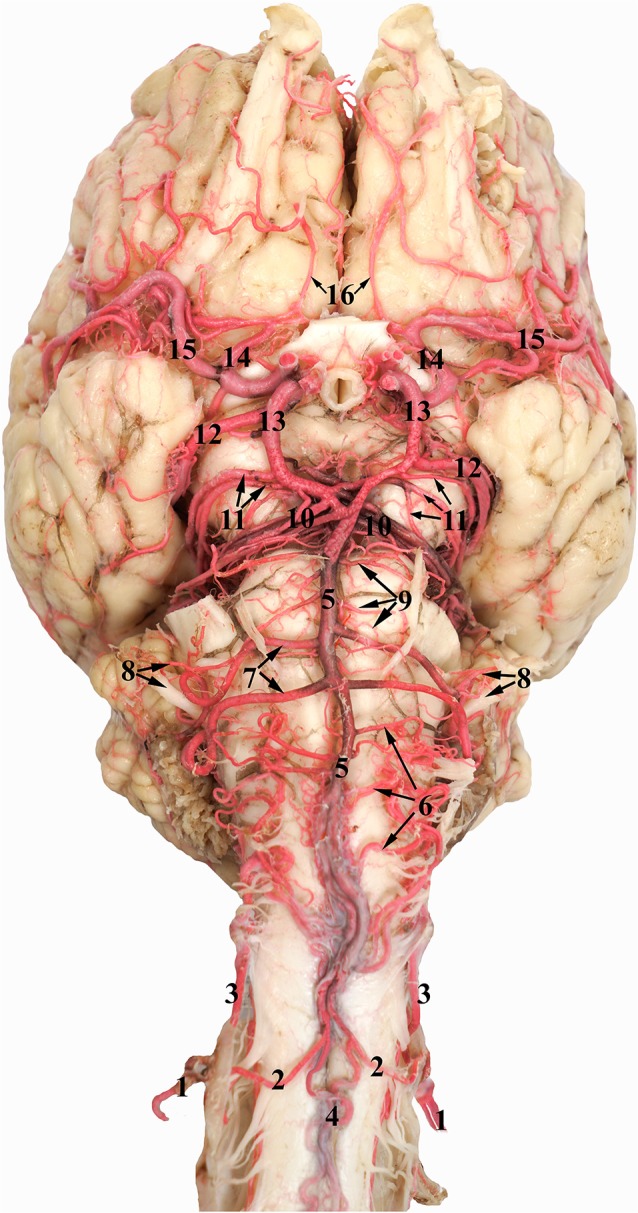
Ventral view of the dromedary brain. 1, Vertebral artery; 2, medial branch of the vertebral artery; 3, lateral branch of the vertebral artery; 4, ventral spinal artery; 5, basilar artery; 6, medullary branches of the basilar artery; 7, caudal cerebellar artery; 8, labyrinthine artery accompanied by the vestibulo-cochlear nerve; 9, pontine branches of the basilar artery; 10, rostral cerebellar artery; 11, caudal choroidal artery; 12, caudal cerebral artery; 13, caudal communicating artery; 14, rostral cerebral artery; 15, middle cerebral artery; and 16 internal ethmoidal artery.

**Figure 7:**
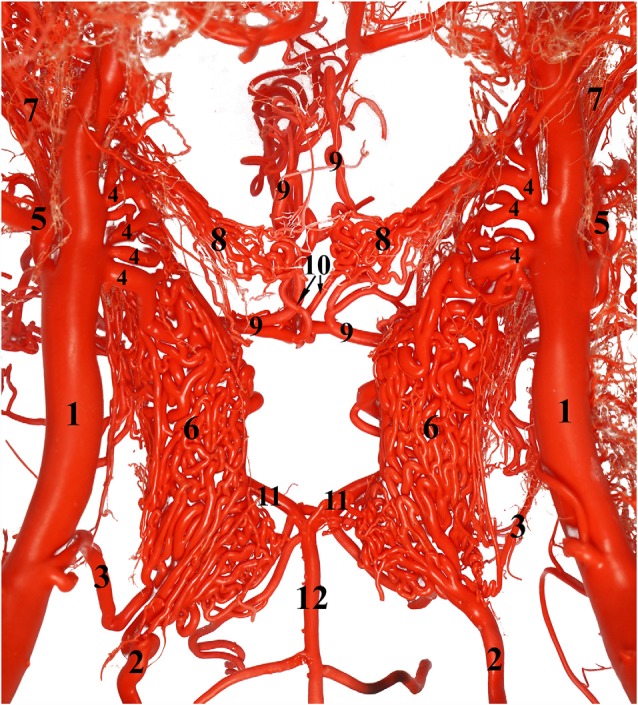
Ventral view of the RERM. 1, Maxillary artery; 2, internal carotid artery; 3, middle meningeal artery; 4, rostral branch to the RERM; 5, external ophthalmic artery; 6, RERM; 7, ophthalmic rete; 8, chiasmatic rete; 9, rostral cerebral artery; 10, internal ophthalmic artery; 11, caudal communicating artery; and 12, basilar artery leading to the cerebral arterial circle. Note: middle meningeal artery was a dominant supplier to RERM in this sample.

Before sending out the rostral branches medially, the maxillary artery sends out the external ophthalmic artery as a lateral branch. The external ophthalmic artery moves in a dorso-lateral fashion towards the orbit and forks out lateral to the maxillary artery (Figures 2, 4, 7). Thereafter, it sends out thick branches that anastomose profusely and contribute to the rostral root of the RERM. These contributions form the dorso-lateral fork of the RERM. After the external ophthalmic artery travels further rostrally, it sends out finer anastomosing branches that form the ophthalmic rete (Figures 4, 7). This extracranial rete sends out several arteries supplying the orbit, lacrimal gland, temporal muscles, retina, olfactory bulb, nasal cavity, and eye muscles. The caudal end of this rete is continuous with the RERM, thus forming the extracranial and rostro-lateral parts of the RERM (Figures 4, 5).

### Middle Meningeal Artery

Our observations have shown that the middle meningeal artery occasionally contributes to the RERM. Among the samples with middle meningeal contribution, the right side was more dominant with a larger artery, than the left side of the rete. When the maxillary artery courses medial to the ramus of the mandible and medial pterygoid muscle, it sends out the middle meningeal artery from its lateral border (Figure 4). We observed variations with the middle meningeal artery emerging from the medial side of the maxillary artery and curving along the ventral aspect to reach the lateral side of the artery before its ascent. This artery rises dorso-medially, crossing the lateral aspect of the tympanic bullae, before entering the oval foramen with the mandibular nerve. After it enters the cranial cavity, it sends out multiple branches that supply the meninges. One of them travels caudally towards the intracranial opening of the foramen ovale, where it bends before dividing into multiple branches and contributing to the caudal root of the RERM. While all specimen showed the branches supplying to the meninges, only a few showed branches contributing to the RERM. The middle meningeal artery joins the rete in close proximity to the internal carotid artery (Figure 3).

### Rostral Epidural Rete Mirabile

The RERM is a dense network of anastomoses of small-to-medium-sized arteries located at the base of the cranial cavity. It is located in the cavernous sinus of the cranial cavity, ventral to the brain, bordered laterally by the trigeminal and abducent nerves and medially by the hypophysis (Figure 5). The two lobes of the rete communicate with each other through a few reciprocating anastomoses, which gives it an H-shaped appearance (Figures 3, 5). This communication occurs at the caudal end along the caudal border of the hypophysis, cranial to the dorsum sellae (Figures 3, 5). The hypophysis is wedged between the two lobes of the RERM. The portion rostral to the hypophysis forms the rostral compartment (rostral root), and the portion caudal to the hypophysis forms the caudal compartment (caudal root) of each lobe of the rete (Figures 3, 5). Interestingly, the rostral end of the rete is forked, with the rostral branches to the RERM from the maxillary artery contributing to the ventral fork. Subsequently, the dorsal fork is composed of anastomosing branches from the external ophthalmic artery (Figure 4C). These forked ends are seen in close proximity of the foramen orbitorotundum, facing the orbital fissure. The forked ends mesh with each other to form the rostral root of the RERM. The caudal root of the rete is chiefly formed by the anastomosing arteries from the internal carotid and middle meningeal arteries. These arteries send out anastomosing branches to the opposite lobe, forming the cross-link in the H-shaped rete. Thus, the paired RERM is the anastomosing end product of the internal carotid artery, middle meningeal artery, rostral branches from the maxillary artery, and contributions from the external ophthalmic artery (Figures 4, 7). This whole structure is embedded in the cavernous sinus and is separated from overlying structures by a thick layer of the dura mater. Only four arteries emerge dorsally from the RERM penetrating the dura mater. They arrive at the base of the brain to form the cerebral arterial circle (Figure 6). These are the right and left rostral cerebral arteries and the right and left caudal communicating arteries (Figures 3, 6, 7).

### Cerebral Arterial Circle

The cerebral arterial circle functions like a ring that collects afferent blood from the RERM and the basilar artery before supplying to the brain. It was previously called the Circle of Willis and is an hourglass-shaped anastomoses of the paired rostral cerebral arteries, paired caudal communicating arteries, and the single basilar artery (Figure 6). It is located in the middle cranial fossa at the base of the brain. It wraps around the optic chiasma, infundibulum of the neurohypophysis, and mammillary body (Figure 6).

The rostral cerebral and caudal communicating arteries arise from the RERM. They pierce the dura mater at the level of the lateral sylvian fissure, lateral to the optic chiasma. Both arise close to each other, but as independent arteries. Their point of origin forms the narrowest point of the hourglass-shaped cerebral arterial circle (Figure 6).

The paired rostral cerebral arteries form the lateral wall of the cerebral arterial circle in the rostral half, while the paired caudal communicating arteries form the lateral wall in the caudal half. The caudal wall of the cerebral arterial circle is sealed by the single basilar artery. This end could either be a simple Y-shaped structure or a more complex triangular/rhomboidal structure formed by the communicating arteries between the two sides (Figure 6). These anastomosing structures are seen in the interpeduncular fossa, between the two oculomotor nerves.

### Rostral Cerebral Artery

The paired rostral cerebral arteries arise directly from the RERM at the medial extremity of the lateral sylvian fissure, lateral to the optic chiasma. After penetrating the dura mater, the rostral cerebral artery sends out very small and short arteries dorsally and forms a plexus when it supplies the hypothalamus. It then sends out the rostral choroidal artery followed by the middle cerebral artery. The rostral cerebral artery passes rostro-medially to the beginning of the longitudinal fissure. Here it anastomoses with its partner on the opposite side *via* a short arterial bridge called the rostral communicating artery. This bridge was absent in most cases, where the two arteries fuse medially before climbing along the longitudinal fissure. The rostral cerebral artery sends out branches to the medial surface of the olfactory and frontal lobes.

Thereafter, the two arteries run rostrally and dorsally in a curved fashion through the longitudinal fissure around the genu of the corpus callosum. At this phase, the rostral cerebral arteries flow caudally on the medial surface of the cerebral hemispheres and produce cortical branches that supply the medial surface of the frontal and parietal lobes of the cerebral hemisphere (Figure 2). These cortical branches are long enough to supply even the dorsal surface of the frontal and parietal lobes.

The rostral choroidal artery is a small branch which arises from the rostral cerebral artery near its origin. It travels laterally and then climbs dorsally between the temporal lobe and cerebral crus and enters the lateral ventricle through the choroidal fissure to end in the choroid plexus of the third and lateral ventricles (Figure 2). The rostral choroidal artery travels close to the caudal cerebral artery during its ascent (Figure 2).

Compared to the RERM, the chiasmatic rete is a smaller mesh of anastomosing arteries that appears to connect the two rostral cerebral arteries to the external ophthalmic artery or rostral branches of maxillary artery. The chiasmatic rete has a medial root of internal ophthalmic artery, which are three or four thicker arteries emerging from the rostral cerebral artery. The lateral root of a few thinner anastomosing arteries from the charismatic rete connects to the external ophthalmic artery within the ophthalmic rete, situated quite close to rostral branches of maxillary artery (Figure 7). The chiasmatic rete is situated at the level of the optic chiasma and is oriented rostro-ventral to the cerebral arterial circle (Figure 7).

After giving out the internal ophthalmic artery, the rostral cerebral artery gives out the internal ethmoidal artery and its lateral branch (Figure 2). These branches climb towards the cribriform plate and run along the olfactory tract (Figure 6). They send out finer branches that curve around and encircle the olfactory tract. They end by giving out finer branches that supplies the ethmoid bone and structures close to the cribriform plate. They also exit the cranial cavity at this region to supply the ethmoturbinates close to the cribriform plate.

### Middle Cerebral Artery

The middle cerebral artery is derived from the rostral cerebral artery rostral to the origin of the choroidal artery. It might have a single point or double points of origin from the rostral cerebral artery. It curves out laterally in front of the pyriform lobe and over the trigeminal nerve. As it reaches the lateral edge of the cerebrum, it climbs dorsally through the lateral sylvian fissure. Here it divides into several branches and supplies to the lateral surface of the frontal, parietal and temporal lobes (Figure 2). These branches dive slightly deeper into the cortical sulci and supply the dorsal and lateral parts of the frontal, parietal and temporal lobes. Unlike the rostral and caudal cerebral arteries, the middle cerebral artery is distinguished by its superficial route around the curvature of the cerebrum. However, the middle cerebral artery also has a few small deep branches that supply the thalamus and basal ganglia.

### Caudal Communicating Artery

After dorsally arising from the RERM, the caudal communicating artery runs caudally in a curved fashion and anastomoses with the basilar artery, wherein they complete and form the caudo-lateral wall of the cerebral arterial circle, connecting the carotid and vertebrobasilar systems. The caudal communicating artery curves around the mammillary body and converges medially in the interpeduncular fossa. It usually sends out thick branches in the caudo-lateral flow and in contrast, thinner branches in the next phase of medial flow (Figure 6). It could send one or multiple branches with varying diameters in the second phase of medial flow. When it reaches the interpeduncular fossa, it anastomoses with the basilar artery, either directly or through multiple anastomosing branches to form a triangular or rhomboidal structure. It also sends out numerous thin branches to form an interpeduncular plexus at this point of convergence to supply the cerebral peduncles of the midbrain (Figure 6).

Arteries arising from the caudal communicating artery can be divided into the cortical and medullary groups. The cortical group supplies to the cortical region of the occipital, parietal, and temporal lobes of the cerebrum. The medullary group supplies to the brainstem and associated structures, deep or ventral to the cerebrum. The artery supplying to the cortex usually comprises one or two thick arteries arising from the caudal communicating artery at its most convex caudal point of the cerebral arterial circle. Usually identified as the caudal cerebral arteries, these are the largest of the arteries emerging from the caudal communicating artery at the root of the oculomotor nerve (Figure 6). They travel laterally and climb up along the curvature of the cerebral peduncle and corpora quadrigemina, close to the rostral choroidal artery from the rostral cerebral artery. They then move medially and come close to their pair dorsal to the quadrigeminal plate of the midbrain towards the longitudinal sulcus. During this phase of the dorso-medial climb, they send out several branches in a caudal direction, that further divide and supply the caudal and medial cortical regions of the occipital lobe. After reaching the longitudinal sulcus, the artery takes a hairpin bend and moves dorsally to produce branches supplying the medial and dorsal surfaces of the occipital and parietal lobes. These branches then curve outward to send branches to the lateral surface of the parietal and occipital lobes.

The caudal choroidal branches, emerging from the caudal communicating artery just before its anastomosis with the basilar artery, are smaller and have multiple origins. They are thin and highly tortuous, with very fine hair-like short branches emerging in a dorsal fashion. These hair-like branches form the interpeduncular plexus at the interpeduncular fossa and supply the cerebral peduncles as the arteries move in a more caudo-lateral fashion. The caudal choroidal branches climb dorsally along the curvature of the cerebellar peduncles to reach the dorsal surface of the brain stem while supplying the caudal and rostral colliculi. They travel to the longitudinal sulcus and anastomose with their pair around the pineal gland. They also send fine branches rostrally to supply the choroid plexuses of the third and lateral ventricles. These branches also supply the thalamus, splenium of the corpus callosum and pineal gland. These tortuous arteries and the cortical branches impart a two-level appearance to the cerebral arterial supply in the caudal half of the brain (Figure 2).

While the caudal cerebral artery and caudal choroidal branches traverse through the caudal surface of the occipital lobe, they move close to the rostral cerebellar artery but do not send communicating or anastomosing branches to them. A clear demarcation between the branches of the caudal communicating artery and rostral cerebellar arteries is evident in the arterial cast (Figures 1, 2).

### Basilar Artery

The basilar artery originates from the fusion of the branches from the vertebral artery. Unlike the other afferent arteries, the basilar artery is usually single and travels along the ventral surface of the medulla oblongata and pons. It runs rostrally, supplying the medulla oblongata, cerebellum, pons and parts of the midbrain (Figure 6). At the level of the interpeduncular fossa, it anastomoses with the caudal communicating artery. It forms the caudal wall of the cerebral arterial circle and shunts a significant volume of blood into the cerebral arterial circle (Figure 6).

The vertebral artery travels through the transverse foramen of the atlas and anastomoses with the occipital artery at the level of the alar foramen. It then passes through the lateral vertebral foramen, opening into the vertebral canal, and sends out a branch which moves cranially along the epidural space without penetrating the dura mater. Inside the vertebral canal, the vertebral artery divides into the lateral and medial branches before penetrating the dura mater. This branching occurs at the level of the alar foramen. The two branches move together rostrally outside the dura mater, piercing it at the level of the foramen magnum. Inside the subarachnoid space, the medial branch moves to the ventral surface of the spinal cord and anastomoses with the ventral spinal artery. The combined branch of the vertebral and ventral spinal arteries moves rostrally to join its pair to form the basilar artery at varying locations at the ventral aspect of the medulla oblongata. The basilar artery travels rostrally along the ventral aspect of the medulla oblongata (Figure 6). The lateral branch of the vertebral artery travels rostrally in the subarachnoid space along the lateral aspect of the spinal cord and ends by anastomosing with the medullary branches of the basilar artery in the caudal cranial fossa (Figure 6).

The basilar artery sends out various characteristically thin and tortuous medullary branches which appear like a mesh around the lateral and dorsal aspects of the medulla oblongata (Figure 6). They anastomose with each other and travel along the curved body of the medulla oblongata to supply throughout its lateral and dorsal aspects. The medullary branches also seem to communicate with the caudal cerebellar artery and the lateral branch of the vertebral artery as mentioned before (Figure 6).

At the rostral end of the medulla oblongata, and the caudal border of the pons, we observed two pairs of prominent arteries arising from the basilar artery. They were the caudal cerebellar arteries, arising perpendicular to the basilar artery at the caudal margin of the pons. The two roots move laterally and fuse at the lateral aspect of the medulla oblongata. Of these, the rostral root sends out the labyrinthine artery that moves laterally into the internal auditory meatus along with the vestibulo-cochlear nerve (Figure 6). Thereafter, the rostral root fuses with the caudal root and both move caudo-laterally, crossing over the abducens nerve and under the facial nerve, before reaching the cerebellum (Figure 6). They supply the choroid plexus of the fourth ventricle before sending out branches to the caudo-ventral aspect of the cerebellum. The caudal cerebellar artery anastomoses with the medullary branches of the basilar artery to supply the medulla oblongata. It also anastomoses with the rostral cerebellar artery (Figure 6).

After sending out the prominent and long caudal cerebellar artery, the basilar artery traverses through the ventral aspect of the pons. The pontine arteries emerge perpendicular to the basilar artery. They are thin, short, evenly spaced, and parallel to each other. These are also seen anastomosing with the caudal cerebellar arteries and rostral cerebellar arteries (Figure 6).

At the rostral border of the pons, the basilar artery bifurcates to form a triangular or rhomboidal structure. Just before this junction, the basilar artery sends out multiple branches in a caudo-lateral direction that could be clubbed together to be called the rostral cerebellar artery. The rostral cerebellar artery includes multiple arteries of varying number, arising chiefly from the basilar artery right before it anastomoses with the caudal communicating artery (Figure 6). The main identifying characteristic of the rostral cerebellar artery is the caudo-lateral direction of flow (Figure 2), crossing the cerebral peduncle and trigeminal nerve before moving along the paravermis to supply the rostral half of the cerebellum.

## Discussion

### Dual Afferent Arterial Systems of Cerebral Circulation

The dromedary brain is thus supplied by two arterial systems: the carotid and vertebrobasilar arterial systems (Schmid et al., 2019). Both systems supply blood to the cerebral arterial circle but differ in the nature of supply.

The carotid arterial system mainly comprises the internal carotid and branches of the maxillary arteries which supply indirectly through the RERM. Cattle, sheep, goat, pigs, carnivores, and camels exhibit this rete also called the carotid rete (Zguigal, 1988; Zguigal and Ghoshal, 1991b; Ocal et al., 1998; Jerbi and Pérez, 2018). This complex network of anastomosing arteries replaces the internal carotid artery and acts as a pooling system, wherein blood from both the arteries mixes and forms a small reservoir of pooled blood (O’Brien and Bourke, 2015). This buffer volume of blood then exits the RERM to form the rostral cerebral and caudal communicating arteries, which form the major contributors and lateral walls of the cerebral arterial circle (García-Villalón et al., 1989). It is widely known as a mechanism for thermoregulation, and baroregulation (Simoens et al., 1987; Zguigal and Ghoshal, 1991a; Samara et al., 2013; Deepthi et al., 2016; O’Brien et al., 2016).

In contrast, the vertebrobasilar arterial system is a supplementary afferent arterial system which supplies directly to the Circle of Willis. The vertebral arteries add to the ventral spinal artery to form the basilar artery, which can be a single or double set of vascular channels that anastomose with the caudal communicating arteries and constitute the caudal wall of the cerebral arterial circle. This arterial system transports blood directly from the subclavian arteries and is pulsatile in nature (Zguigal and Ghoshal, 1991b; Wake-Buck et al., 2012). Unlike the RERM, this channel is a direct connection from the heart and forms a significant supply to the brain.

### Anastomoses and Bypasses

The blood reaching through the carotid and vertebrobasilar systems requires several supplementary shunts and bypasses. Although the rete is the major interphase between blood flowing from the carotid arterial system to the cerebral arterial circle, there are some direct connections that bypass the rete. The first prominent shunt is the occipital artery that branches out from the common carotid artery at the level of the atlas and contributes to the vertebral artery. The occipital artery, after its origin, moves lateral to the internal carotid artery and vagus nerve, caudo-dorsally along the atlantal fossa and enters the alar foramen of the atlas, where it anastomoses with the vertebral artery that travels through the transverse foramen. This occipital-vertebral anastomosis has been widely studied in all species (Daniel et al., 1953; Baldwin and Bell, 1963; Zguigal, 1988; Cranley, 2011; Deepthi et al., 2016). In this study, we found that after this anastomosis, the vertebral artery sends out major muscular branches that supply the neck muscles. The medial branches of the vertebral arteries contribute to the ventral spinal arteries to form the basilar artery.

The second connection occurs between rostral cerebral artery and external ophthalmic artery, known as the chiasmatic rete (Figure 7). Chiasmatic rete has been described in camelids, sheep, and bovines (Daniel et al., 1953; Hseih and Takemura, 1994; Zdun et al., 2013; Kiełtyka-Kurc et al., 2014). It is a small anastomosis of fine arteries located near the optic chiasma. We found that the rostral cerebral artery sends out thin internal ophthalmic arterial branches to the chiasmatic rete just before it begins its ascent. This rete has connecting arteries to the ophthalmic rete and sometimes very close to the rostral branches to the RERM of the maxillary artery (Figure 7). Kiełtyka-Kurc et al. (2014) also described its connection, but with the external ethmoidal artery in camels. In the same location, other authors identified the thin artery associated with the chiasmatic rete as the internal ophthalmic artery in bovines and camels (Zguigal and Ghoshal, 1991b; Zdun et al., 2013; Kiełtyka-Kurc et al., 2014). Our observations hint that the external ethmoidal artery, passes through the multiple ethmoid foramen and gives out the tortuous rostral meningeal artery. The rostral meningeal artery supplies the meninges in the ethmoid fossa and dorsal aspect of the frontal region and thus, having no direct connection with the chiasmatic rete in the dromedary. The chiasmatic rete is known to regulate the temperature in the orbital region (García-Villalón et al., 1989; O’Brien, 2018).

We have also observed a clear disconnect between the internal ethmoidal artery (arising from the rostral cerebral artery) and the external ethmoidal artery (passing through ethmoid foramen). This suggests that in camels, the external ethmoidal artery does not contribute to the Rostral Epidural Reta Mirabile.

At the level of the chiasmatic rete, only few samples showed the rostral communicating artery connecting between the two rostral cerebral arteries. This is similar to the observation made by Kiełtyka-Kurc et al. (2014) in Bactrian camels. However, the rostral communicating artery is a dominant variation in bovines and other camelids like Llamas (Zdun et al., 2013; Kiełtyka-Kurc et al., 2014).

The third arterial shunt is seen between the vertebral and basilar arteries. After its anastomosis with the occipital artery, the vertebral artery sends out a medial branch that contributes to the ventral spinal artery. It also sends out a lateral branch that travels along the lateral aspect of the spinal cord. It ends by anastomosing with the medullary branches of the basilar artery. This shunt could play a role in maintaining blood pressure and blood flow in long-necked dromedary when they raise or lower their heads (García-Villalón et al., 1989).

### Internal Carotid Artery

Some authors consider the blood vessels emerging from the rete as the intracranial portion of the internal carotid artery. This is more evident in bovine, yak, goat, sheep, and pig, in which the extracranial part of the internal carotid artery exists in the ontogenesis of the rete and contributes to its formation before regressing in the later stages of gestation (Daniel et al., 1953; Kapoor et al., 2003; Cranley, 2011; Kiełtyka-Kurc et al., 2015; O’Brien et al., 2016). In these species, the maxillary and external ophthalmic arteries actively contribute to the RERM (Baldwin and Bell, 1963; Zdun et al., 2013; Jerbi et al., 2016). We have not found any direct connection between the internal carotid artery and arteries emerging from the RERM. This observation was also made by Kiełtyka-Kurc et al. (2014) in camels. El Allali et al. (2017) describes the entry of carotid artery through an opening on the tympano-occipital fissure, termed as carotid fissure by Lesbre, 1903). Our observations and illustrations support this. This entrance is different from that found in the dog, where the internal carotid artery enters through lacerate foramen (Evans and Miller, 2013).

### Middle Meningeal Artery

Authors describing arteries contributing to the RERM in camels have often overlooked the contribution of the middle meningeal artery (Ocal et al., 1999; Kiełtyka-Kurc et al., 2014; Jerbi et al., 2016). However, Zguigal (1988) mentioned its contribution to the RERM at its caudal root in camels (Zguigal and Ghoshal, 1991b). Long-necked animals, like the giraffe, dog, and cat species also exhibit a middle meningeal artery that contributes to the RERM (Daniel et al., 1953; Kanan, 1970; Simoens et al., 1987; Kapoor et al., 2003; Parkash and Jain, 2014; O’Brien et al., 2016). We were able to locate this contribution in the chemically digested arterial casts and less successfully in the dissected samples. This loss of detail during dissection could be attributed to the location of the middle meningeal artery at its point of origin. It travels in close proximity and medial to pterygoid muscles and ramus of the mandible and is at a high risk of destruction when removing the mandible during dissection. The diameter of this artery also varies across different samples and between the left and right arteries. This marked variation in thickness raises questions regarding the functionality of this contribution. In camels, the middle meningeal artery enters cranial cavity through foramen ovale, similar to the ramus caudalis ad rabile epidurale rostrale seen in ruminants (Simoens et al., 1987; Fukuta et al., 2007; Zdun et al., 2013). All our samples showed middle meningeal artery with dominant meningeal branches contributing to the meninges. In addition to these meningeal branches, they had contributory branch to the RERM of varying thickness.

### Rostral Cerebellar Artery

The basilar-caudal communicating anastomosis is so close to the point of origin of the rostral cerebellar arteries that some animals show multiple origins of the rostral cerebellar artery. In some animals, the rostral cerebellar arteries are mistaken to originate from the caudal communicating arteries. The arteries originating from the caudal communicating artery, namely, the caudal cerebral and caudal choroidal arteries, travel up and supply the cerebrum. In contrast, the rostral cerebellar arteries, which originate from the basilar artery or from its junction, travel caudally to supply the cerebellum. The course of flow of the rostral cerebellar artery helps distinguish it from the neighboring caudal choroidal artery. Although this study highlights multiple points of origin of the caudal cerebral artery, similar arteries have previously been identified as the rostral cerebellar artery (Kiełtyka-Kurc et al., 2015). Khairuddin et al. (2017) observed this distinction between the caudal cerebral and rostral cerebellar arteries in donkeys. The rostral cerebellar artery is a branch of the caudal cerebral artery in dogs, rabbits, goats, and sheep. Only monkeys have the rostral cerebellar artery exclusively emerging from the basilar artery (Kapoor et al., 2003). We can thus conclude that camels also have a distinct basilar artery exclusively sending out branches that supply the cerebellum, i.e., the rostral and caudal cerebellar arteries.

### Disconnect Between the Caudal Cerebral and Rostral Cerebellar Arteries

While the rostral cerebellar and caudal cerebral arteries lie close at their point of origin, they move in different directions and supply different regions of the camel brain. In this study, the epoxy cast models of both the arteries showed a clear demarcation between the region of supply of both the arteries. They do not seem to anastomose with each other, thus contradicting previous observations that hint at communicating channels between the two arteries (Evans and Miller, 2013; Kiełtyka-Kurc et al., 2014).

In conclusion, the afferent blood reaches the brain through two dominant arterial systems, the carotid and vertebrobasilar systems. Despite wide variations, arterial vascular architecture of the camel brain is similar to those of other artiodactyls in exhibiting multiple sources of oxygenated blood to the cerebral structures and multiple levels of blood pooling between the different sources. Shunts like those from the lateral branch of the vertebral artery and retia like the chiasmatic rete warrant further studies on their anatomical position and functional role.

### Labyrinthine Artery

Similar to the rostral cerebellar artery, the caudal cerebellar artery shows multiple points of origin from the basilar artery at the caudal border of the pons. This is not common in all camels, despite being a recurring aberration. The labyrinthine artery is a small branch from the rostral root of the caudal cerebellar artery. Kanan (1970) and Salomon et al. (2008) documented these arteries supplying to the internal ear. While this is a direct branch of the basilar artery in dogs (Evans and Miller, 2013), it is just a minor branch of the caudal cerebellar artery in the dromedary.

This study was able to identify often ignored arterial sources to the RERM like the middle meningeal artery. It was able to account for different variations owing to its larger sample size. In addition, this study was able to illustrate the disconnect between cerebral and cerebellar circulation as well as origin of basilar artery. It acts as a primer for further exploration of the various arterial circuits to the camel brain, further highlighting the anatomical uniqueness of dromedary.

## Data Availability

The raw data supporting the conclusions of this manuscript will be made available by the authors, without undue reservation, to any qualified researcher.

## Ethics Statement

This study was carried out in accordance with the recommendations of United Arab Emirates University (UAEU) Research Ethics Committee. The protocol was approved by the Animal Research Ethics Committee (A-REC) at the United Arab Emirates University (ERA_2019_5850).

## Author Contributions

AAA: study design/conception, anatomical dissection, analysis/acquisition of data, photography and photo editing, drafting of the manuscript, and revision of the manuscript. PM: study design/conception, anatomical dissection, analysis/acquisition of data, drafting of the manuscript, and revision of the manuscript. AAD: specimen collection, specimen preparation and anatomical dissection. FA, SA and AB: anatomical dissection and specimen preparation. MQ: correction and revision of manuscript. SS: guidance, correction and revision of manuscript.

## Conflict of Interest Statement

The authors declare that the research was conducted in the absence of any commercial or financial relationships that could be construed as a potential conflict of interest.
